# TRABD maintains mitochondrial homeostasis and protects against ischemia reperfusion-induced renal tubular injury

**DOI:** 10.3389/fcell.2025.1619339

**Published:** 2025-07-24

**Authors:** Wenqi Duan, Wenye Wu, Cui Yang, Mei Zhang, Xuemei Li, Wenmin Tian, Yang Chen, Xinjun Zhang

**Affiliations:** ^1^ Key Laboratory of Molecular Biophysics of the Ministry of Education, College of Life Science and Technology, Huazhong University of Science and Technology, Wuhan, China; ^2^ Research Unit for Blindness Prevention of the Chinese Academy of Medical Sciences (2019RU026), Sichuan Academy of Medical Sciences and Sichuan Provincial People’s Hospital, Chengdu, China; ^3^ Center for Precision Medicine Multi-Omics Research, Institute of Advanced Clinical Medicine, Peking University, Beijing, China; ^4^ Department of Biochemistry and Biophysics, School of Basic Medical Sciences, Peking University Health Science Center, Beijing, China; ^5^ The Key Laboratory for Human Disease Gene Study of Sichuan Province and the Department of Laboratory Medicine, Sichuan Provincial People’s Hospital, University of Electronic Science and Technology of China, Chengdu, China

**Keywords:** TRABD, mitochondrial membrane protein, dynamics, mitophagy, PGAM5, ischemia reperfusion-induced renal tubular injury

## Abstract

Mitochondria serve as hubs for many critical cellular processes, and their functions and dynamics are tightly controlled. TRABD is a Tiki/TraB family protein with unknown function. Here, we characterized TRABD as a novel outer mitochondrial membrane protein. Depletion of *TRABD* in cells severely impairs mitochondrial respiration and ATP production, inhibits cell growth, increases reactive oxygen species levels. Depletion of *TRABD* also affects mitochondrial dynamics and mitophagy, possibly through interactions with PGAM5. Knockout of *TRABD* in mice significantly exacerbates ischemia reperfusion-induced renal tubular injury by promoting mitochondrial fragmentation and damage. Our study identified a novel outer mitochondrial membrane protein and revealed the critical roles of TRABD in mitochondrial dynamics and ischemia reperfusion-induced renal tubular injury.

## 1 Introduction

Mitochondria serve as hubs for many cellular processes, including bioenergetics, metabolism, cellular signaling, redox balance, calcium homeostasis, and cell death (Wai et al., 2016). Mitochondria are highly dynamic, double-membraned organelles that constantly undergo fusion and fission. Mitochondrial dynamics is a general term that describes mitochondrial changes, including mitochondrial fusion, mitochondrial fission, and mitochondrial trafficking (Chen et al., 2023). Among the numerous molecules that regulate mitochondrial dynamics, dynamin-related protein 1 (DRP1) and phosphoglycerate mutase (PGAM5) play important roles. Mitochondrial fission is promoted by DRP1, which localizes to the mitochondrial membrane via adaptor proteins and drives fission in a GTP-dependent manner (Losón et al., 2013). DRP1 GTPase activity can be reduced by DRP1 phosphorylation at S637, while DRP1 phosphorylation at Ser616 promotes mitochondrial fission by recruiting more DRP1 to the mitochondrial surface (Giacomello et al., 2020). PGAM5 affects mitochondrial morphology, motility, and mitophagy and regulates multiple programmed cell death. However, the effects of PGAM5 on mitochondrial morphology are still controversial (Cheng et al., 2021).

Changes in mitochondrial dynamics are closely related to mitophagy and cell death (Ma et al., 2020). In the case of energetic disbalance and dysfunctional or depolarized mitochondria, the mitochondrial membrane potential dissipates, mitochondrial fission increases, and damaged mitochondria are removed specifically by mitophagy. As a mechanism of mitochondrial quality control, mitophagy maintains cell fitness, preventing cellular damage (Chen et al., 2016; Yamashita and Kanki, 2017). However, mitochondrial fission may also facilitate apoptosis in response to severe cellular stress (Cereghetti et al., 2010).

Mitochondrial dynamics are critical for sustaining bioenergetics, particularly in metabolically demanding tissues such as the kidney (Bhargava and Schnellmann, 2017). Disruption of mitochondrial morphology plays an important role in maladaptive kidney repair. Mitochondrial fragmentation resulting from fission activation and fusion arrest has been implicated as a key event in mitochondrial damage and kidney tubule injury (Tang et al., 2021). Fragmented mitochondria are potential sources of ROS, cytochrome c, mitochondrial DNA and other potentially injurious molecules. In animal models of ischemia‒reperfusion (I/R), excessive mitochondrial fragmentation occurs before tubular cell apoptosis, and inhibition of fission attenuates tubular cell death and kidney injury (Brooks et al., 2009). Consistent with these findings, proximal tubule-specific deletion of Drp1 protected mice against I/R-induced renal tubular cell death, inflammation and injury and accelerated kidney recovery (Perry et al., 2018). Optic atrophy 1 (OPA1) is a fusion protein for the mitochondrial inner membrane in mammalian cells that is regulated by proteolytic processing. Overlapping activity with m-AAA protease-1 (OMA1), a zinc metalloprotease, is responsible for OPA1 proteolysis and inactivation during cellular stress. There are reports of OMA1 activation and OPA1 proteolysis in mice following ischemic reperfusion injury. Following I/R, OPA1 proteolysis, mitochondrial fragmentation and kidney injury are attenuated in OMA1-deficient mice compared with wild-type mice (Xiao et al., 2014). Mitochondrial fragmentation and swelling in kidney tubular cells might reduce energy metabolism and increase ROS formation, which could promote tissue damage, inflammation, and maladaptive kidney repair (Emma et al., 2016). However, the precise mechanisms that underlie the deleterious effects of mitochondrial fragmentation on kidney repair await in-depth investigation.

TRABD (TraB domain-containing) belongs to the Tiki/TraB superfamily and is highly conserved in metazoans (Bazan et al., 2013). Tiki proteins are membrane-tethered metalloproteases that hydrolyze the amino terminus of certain Wnt ligands, inhibiting their activity (Zhang et al., 2012). Studies have shown that TRABD promotes fusion through interactions with MFN2, MIGA2, and PLD6. In *Drosophila*, dTRABD deficiency affects muscle mitochondria and motor function, with the effects worsening with age. Overexpression of TRABD exacerbates tau-induced neurodegeneration, suggesting that TRABD-related mitochondrial dysfunction contributes to age-related neurodegenerative diseases (Zhou et al., 2024). However, the precise structure and functions of TRABD have yet to be fully elucidated. Additionally, its regulatory role in non-neuronal diseases in mammals, such as kidney injury, remains poorly understood.

In this study, we also characterize TRABD as an outer mitochondrial membrane protein that plays a critical role in regulating mitochondrial dynamics and function, possibly through interactions with PGAM5. Furthermore, we reveal a protective role for TRABD in I/R-induced renal tubular injury using TRABD knockout mice, highlighting its importance in non-neuronal diseases.

## 2 Materials and methods

### 2.1 Animals

To generate the *TRABD* knockout mouse model on a C57BL/6J background, we employed CRISPR/Cas9-mediated genome engineering. The target region encompassed exons 2–10 of the *TRABD* gene (NCBI RefSeq: NM_026485; Ensembl transcript: ENSMUST00000081702), spanning approximately 5K base pairs and covering 100% of the coding sequence (from the ATG start codon in exon 2 to the TAG stop codon in exon 10). Two single-guide RNAs (sgRNAs) were designed to flank this region: m*TRABD*-sgRNA1 (target sequence: 5′-CCCAGCGGCCTAGTTCCAAAAGG-3′) and m*TRABD*-sgRNA2 (target sequence: 5′- CCTGTTGTAAGGGTCAAGACTGG-3′). These sgRNAs were designed to ensure deletion of the entire coding sequence while minimizing potential off-target effects in the nonoverlapping genomic locus on chromosome 15. Cas9 mRNA and sgRNAs were coinjected into fertilized mouse zygotes, which were subsequently transferred into pseudopregnant female mice. Founder pups were genotyped via PCR amplification of the targeted locus, followed by Sanger sequencing to verify the deletion. This strategy ensured complete ablation of *TRABD* function without disrupting adjacent genes, as the knockout region was confirmed to lack overlapping annotated genes. All animal procedures and experiments were approved by the Animal Care Committee of Huazhong University of Science and Technology and conducted according to the guidelines of the committee. The mice were maintained under a 12-h light/dark cycle at room temperature (22°C–25°C) and 55% ± 5% humidity with *ad libitum* access to water and a standard laboratory diet. The water and cages were autoclaved, and no mice became severely ill until the end of the experiments.

### 2.2 Cell line

HeLa (CRM-CCL-2) and HEK293T (CRL-11,268) cells from the American Type Culture Collection (ATCC) and HEK293A (Procell, CL-0003) cells were cultured in high-glucose Dulbecco’s modified Eagle’s medium (DMEM) supplemented with 10% fetal bovine serum (BioChannel Biological Technology Co., Ltd., BC-SE-FBS07), 2 mM L-glutamine, 100 U/mL penicillin and 100 μg/mL streptomycin at 37°C in a humidified incubator with 5% CO_2_.

### 2.3 Generation of CRISPR-Cas9 knockout cell lines

The human *TRABD*-guide sequence (5′-CGGGAGCAGGGTGTACGTGG-3′) was inserted into the lentiCRISPR v2 plasmid (Addgene, 52,961; deposited by Feng Zhang) via the BsmBI restriction site. Lentiviral particle production was initiated by cotransfecting the plasmid DNA with the lentiviral packaging vectors psPAX2 (Addgene, 12,260; deposited by Didier Trono) and pMD2. G (Addgene, 12,259; deposited by Didier Trono) into HEK293T cells. The lentiviral particles were harvested on days 2 and 3 posttransfection. To generate the knockout cell pools, HEK293A cells were infected overnight with the harvested lentivirus and then selected with 2 μg/mL puromycin. After 2 days, the cells were trypsinized and seeded into a 96-well plate via the limiting dilution method. Finally, monoclonal cells were selected for immunoblotting analysis.

### 2.4 Subcellular fractionation

The cells were collected and resuspended in hypotonic buffer. After gentle homogenization with 26 G needles on ice, the cell lysates were subjected to differential centrifugation and gradient centrifugation. The resulting fractions were lysed and analyzed via Western blotting (Annunziata et al., 2015; Wieckowski et al., 2009).

### 2.5 Protease protection assay

For trypsin digestion, isolated mitochondria were suspended in hypotonic buffer and incubated on ice with 20% trypsin containing the indicated concentrations of 1% Triton X-100 for 30 min. Mitochondrial proteins were separated via SDS–PAGE, and proteins were detected via Western blotting with the indicated antibodies.

### 2.6 Mitochondrial oxygen consumption rate (OCR) assay

O_2_ consumption in living cells was measured via an O2k-FluoRespirometer (O2k, Oroboros Instruments, Innsbruck, Austria). The temperature was kept constant at 37°C ± 0.002°C under constant stirring at 750 rpm, which ensured a homogenous O_2_ concentration in the experimental closed chambers with volumes of 2 mL each. The O_2_ concentration and O_2_ flow per cell were monitored in real time via DatLab 7.4 software (Oroboros Instruments, Innsbruck, Austria). Calibrations of the polarographic O_2_ sensors of O2k were performed daily, and the partial O_2_ pressure was corrected for barometric pressure monitored by O2k.

The addition of cells into the O2k chambers was performed by completely replacing the volume of the sample. First, routine respiration is a physiological coupling state in which respiration is controlled by aerobic ATP demand and coupling efficiency. This step was followed by the inhibition of the phosphorylation system with oligomycin Omy, which induces leaked respiration. The protonmotive force was subsequently dissipated via stepwise titration of the uncoupler U carbonyl cyanide 3-chlorophenylhydrazone (CCCP) until the maximum noncoupled respiration was obtained. Finally, the addition of antimycin A (Ama) inhibited the electron transport pathway, allowing the measurement of residual oxygen consumption. Data analysis was performed via DatLab 7.4.

### 2.7 Mitochondrial extracellular acidification rate (ECAR) assay

Glycolytic function was assessed via a pH ISE module of the O2k-FluoRespirometer (O2k, Oroboros Instruments, Innsbruck, Austria) following the manufacturer’s instructions for the glycolysis assay. Initially, standard pH calibration solutions were used to calibrate the instrument. The cells were subsequently collected, resuspended in pH buffer (2 mM imidazole, 2.4 mM KCl, 0.49 mM MgCl_2_-6H_2_O, 0.42 mM CaCl_2_-2H_2_O, and 137 mM NaCl, pH 7.1 (adjusted at 30°C)), and counted. The cells were then added to the cell compartment, and reference and detection electrodes were inserted for measurement. Reagents were titrated in the following sequence: cells, glucose, the ATP synthase inhibitor oligomycin, and 2-deoxy-D-glucose (2-DG), with each subsequent reagent added dropwise after stabilization of the curve. Data analysis was performed via DatLab 7.4.

### 2.8 Measurement of ATP

A firefly luciferase-based ATP assay kit (Beyotime, S0026) was used to measure the ATP content in the cells according to the manufacturer’s instructions. The cell samples were lysed in lysis buffer and centrifuged. The supernatants were mixed with ATP detection working solution in a white 96-well plate. Standard curves were also generated, and the protein concentration in each group was determined via the BCA protein assay. Total ATP levels are expressed as nmol/mg protein.

### 2.9 Measurement of reactive oxygen species (ROS)

For cytosolic ROS detection, the cells were incubated with 10 μM DCFH-DA (Beyotime, S0033S) at 37°C for 15 min. The cells were then washed with PBS and digested with trypsin. The cells (1 × 10^6^) were collected via centrifugation. ROS were measured via flow cytometry (NovoCyte Quanteon, Agilent, United States), and ROS levels were evaluated via FlowJo software.

### 2.10 Measurement of the mitochondrial membrane potential (Δψm)

For the Δψm assay, treated cells were loaded with the potentiometric dye 500 nM TMRE (Beyotime, C2001S) at 37°C for 20 min. The cells were then washed once with PBS and digested with trypsin. The cells (1 × 10^6^) were collected via centrifugation. The Δψm was measured via flow cytometry (NovoCyte Quanteon, Agilent, United States), and the Δψm was evaluated via FlowJo software.

### 2.11 Immunoprecipitation and Western blotting

The cell pellets were homogenized in lysis buffer containing 20 mM Tris-HCl, 150 mM NaCl, 0.5% NP-40 (Solarbio, N8030), 10% glycerol, protease cocktail (TargetMol, C0001), and phosphatase cocktail (TargetMol, C0002), pH 7.4, and incubated on ice for 30 min. The cell lysate was cleared by centrifugation at 21,130 × g for 10 min. The supernatant was incubated with antibody-conjugated beads and rotated for 12 h at 4°C. After incubation, the beads were washed 5 times with the same lysis buffer. Western blotting was performed following standard procedures.

### 2.12 Immunofluorescence

The cells were grown on coverslips (NEST Biotechnology, 801,010) overnight, fixed in 4% paraformaldehyde (Sangon Biotech, E672002) in PBS for 15 min at room temperature and permeabilized with 0.1% Triton X-100 (Solarbio, T8200) in PBS for 15 min. Following permeabilization, the cells were treated with blocking buffer (10% normal donkey serum (Yeasen Biotechnology, 36116ES03)) for 1 h at room temperature. The cells were incubated with primary antibodies diluted in blocking buffer overnight at 4°C. The cells were washed three times with PBS for 10 min each, followed by incubation with an Alexa Fluor-conjugated secondary antibody in blocking buffer for 1 h at room temperature. Finally, the cells were washed three times with PBS again and stained with DAPI (1 μg/mL). The slides were examined via a laser scanning confocal microscope (Olympus FV3000).

### 2.13 Cell proliferation assays

To evaluate cell proliferation, a Cell Counting Kit-8 (CCK-8) assay (Abbkine, Wuhan, China, BMU106-CN) was used according to the manufacturer’s instructions. In brief, cells were plated in 96-well plates in quintuplicate at a concentration of 1,000 cells per well. At the indicated time points, 10 μL of CCK-8 solution was added, and the plate was returned to the incubator for another 2 h. Then, the OD450 of each well was recorded with a microplate reader.

### 2.14 Cell cycle analysis

The cells were seeded on six-well plates at 37°C for 24 h. Then, the cells were washed with PBS and digested with trypsin. The cells (1 × 10^6^) were collected via centrifugation, washed twice with ice-cold PBS, fixed with cold 70% ethanol and stored at −20°C for 24 h. The cells were subsequently centrifuged again, washed twice with cold PBS, incubated with RNase A (0.1 mg/mL) for 1 h at 37°C, and stained with PI (0.1 mg/mL) for 30 min in the dark. The DNA content was measured via flow cytometry (NovoCyte Quanteon, Agilent, United States), and the percentage of cells in each phase of the cell cycle was evaluated via FlowJo software.

### 2.15 Quantitative reverse transcription‒PCR (qRT‒PCR)

RNA was extracted via an RNA Isolation Kit (Vazyme Biotech Co., Ltd., RC101), and cDNA was obtained via an RT Reagent Kit (Vazyme Biotech Co., Ltd., R323). qRT‒PCR was performed with SYBR Green PCR Master Mix (Tsingke Biotech Co., Ltd., TSE401) via a CFX Connect Real-Time PCR Detection System (Bio-Rad). The experiments were performed using three independent RNAs. The qRT‒PCR results were normalized to those of GAPDH and calculated following the 2^−ΔΔCT^ method.

### 2.16 Mouse proximal tubular epithelial cells isolation

Mouse proximal tubular epithelial cells (PTECs) were isolated from the renal cortex of C57BL/6 mice (Wang et al., 2022; Xiong et al., 2022). The minced renal cortex was digested in 1 mg/mL type II collagenase, and then the digestion was terminated with culture medium. The cells were filtered through a cell strainer (70 μm, Biofil). After being washed with PBS, the precipitate was resuspended in DMEM/F12 (1:1) medium containing 10% FBS for 4‒5 days.

### 2.17 Ischemia‒reperfusion surgery

Renal unilateral ischemia‒reperfusion (UIRI) was induced as previously reported (Lee et al., 2024). Briefly, the mice were anesthetized via an intraperitoneal injection of 1% pentobarbital and placed on a warming pad to maintain body temperature at 37°C ± 0.3°C. The left renal vessels (vein and artery) were exposed and clamped with a vascular clip (Roboz Surgical Instrument Co., Germany) for 30 min. The clamps were then released to allow reperfusion to the kidney. The sham-operated mice underwent the same procedure, apart from vessel clamping. The mice were euthanized at 1, 3, and 7 days post-UIRI, and blood and kidney samples were collected for further analyses.

### 2.18 Renal tubular injury score evaluation

For histological analysis, the paraffin-embedded kidney was sliced into 4-µm sections, which were subjected to periodic acid-Schiff staining (G1281, Solarbio, China) according to the manufacturer’s instructions. At least 10 fields viewed via a microscope were randomly selected. Kidney injury was scored in a blinded manner according to the percentage of injured renal tubules and histological injury, which was indicated by brush border loss, tubular dilation/flattening, tubular degeneration, tubular cast formation, and vacuolization.

### 2.19 TUNEL staining

The DeadEnd^™^ Fluorometric TUNEL System is a classic TUNEL assay designed for the specific detection and quantification of apoptotic cells within a cell population (G3250, Promega, United States). Paraffin-embedded tissue sections were first deparaffinized by washing in xylene and rehydrated through decreasing ethanol concentrations. After fixation in 4% formaldehyde and permeabilization with Proteinase K, the tissue was equilibrated with buffer. The TdT reaction mix was then applied, and the slides were incubated at 37°C for 60 min in the dark. The reaction was stopped with 2X SSC, and the slides were washed in PBS. After mounting with a medium containing DAPI for counterstaining, apoptotic cells were visualized under confocal fluorescence microscopy, showing green fluorescence for apoptotic cells and blue for DAPI-stained nuclei.

### 2.20 Quantification and statistical analysis

Statistical analyses were performed using GraphPad Prism v9.0. Data were compared using Student’s t-test and are presented as means ± SEM, unless otherwise noted. Statistical tests are specified in the corresponding figure legends. Significant differences are indicated as follows: *P < 0.05, **P < 0.01, ***P < 0.001, and ****P < 0.0001.

## 3 Results

### 3.1 TRABD is a mitochondrial outer membrane protein

**FIGURE 1:**
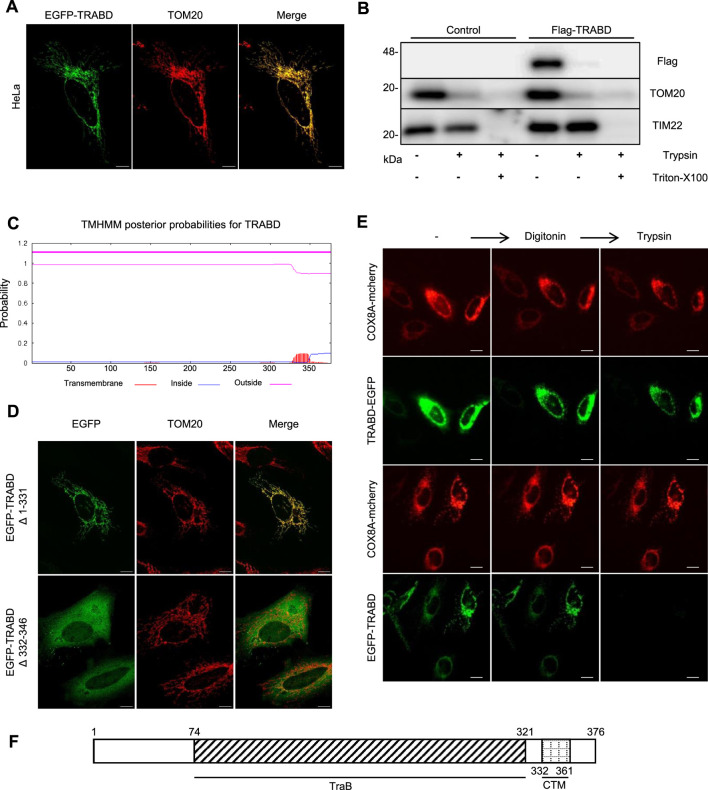
TRABD is a mitochondrial outer membrane protein. **(A)** HeLa cells expressing EGFP-TRABD were immunostained with an anti-TOM20 antibody. Note that EGFP-TRABD is perfectly colocalized with TOM20. (Scale bar, 10 μm). **(B)** Control 293T cells or 293T cells expressing Flag-TRABD were homogenized and fractionated. The mitochondrial fractions were digested with trypsin with or without Triton X-100 and analyzed by immunoblotting with the indicated antibodies. **(C)** Hydrophobicity plot for TRABD predicted by TMHMM-2.0, showing a possible transmembrane domain on the carboxyl terminus of TRABD. **(D)** The predicted transmembrane domain of TRABD is necessary and sufficient for mitochondrial localization. HeLa cells expressing EGFP-TRABD (Δ1-331) or EGFP-TRABD (Δ332-346) were fixed, immunostained with an anti-TOM20 antibody and visualized via confocal microscopy. (Scale bar, 10 μm). **(E)** TRABD is an outer mitochondrial membrane protein with its amino terminus positioned outside and its carboxyl terminus positioned inside the mitochondria. HeLa cells expressing TRABD-EGFP or EGFP-TRABD together with COX8A-mCherry were permeabilized with 20 mM digitonin, and live cell images were taken before and after 1 min of treatment with 4 mM trypsin (scale bar, 20 μm). **(F)** Schematic representation of the TRABD protein showing the TraB homology domain and a carboxyl-terminal transmembrane domain (CTM).

To study the function of TRABD, we first examined its subcellular localization. Immunofluorescence revealed that the EGFP-TRABD fusion protein expressed in HeLa cells colocalized perfectly with the mitochondrial outer membrane protein TOM20 (Figure 1A). We found that the tagging position of TRABD affects its subcellular localization. C-terminal tagging causes partial diffusion in the cytosol, while N-terminal tagging results in complete co-localization with TOM20 (Supplementary Figure S1A–C). Therefore, all subsequent functional experiments used N-terminal tagged TRABD to ensure precise localization. We further isolated crude mitochondria and performed a protease protection assay with or without the detergent Triton X-100. The results indicated that FLAG-TRABD is enriched in the mitochondrial fraction and efficiently digested by trypsin in the absence of Triton X-100, suggesting that TRABD is localized on the surface of mitochondria (Figure 1B). In addition, a secondary structural prediction of TRABD revealed a putative transmembrane domain located at its carboxyl terminus (Figure 1C). We further showed that this predicted transmembrane domain is both necessary and sufficient for TRABD localization to mitochondria, as deletion of this region (EGFP-TRABD△332-346) abolished the mitochondrial localization of EGFP-TRABD, and this region fused with EGFP (EGFP-TRABD△1-331) was sufficient to target EGFP to mitochondria (Figure 1D). The precise transmembrane localization of TRABD was further demonstrated (Supplementary Figure S1D–F). To further analyze the topology of TRABD on mitochondria, we performed a fluorescence protease protection assay. Live cells coexpressing TRABD-EGFP (EGFP was added to the carboxyl terminus of TRABD) or EGFP-TRABD (EGFP was fused to the amino terminus of TRABD) and COX8A-mCherry (a mitochondrial matrix protein) were first incubated with digitonin, which permeabilizes the plasma membrane but not the mitochondrial outer membrane and then incubated with trypsin solution. The results revealed that trypsin diminished the EGFP signal in EGFP-TRABD-expressing cells but had no effect on the EGFP signal in TRABD-EGFP-expressing cells, further suggesting that TRABD is an outer mitochondrial membrane protein whose amino terminus is exposed on the surface of mitochondria (Figure 1E). Collectively, these results demonstrate that TRABD is anchored to the mitochondrial outer membrane through a C-terminal transmembrane domain (Figure 1F).

### 3.2 *TRABD* knockout perturbs mitochondrial function

**FIGURE 2:**
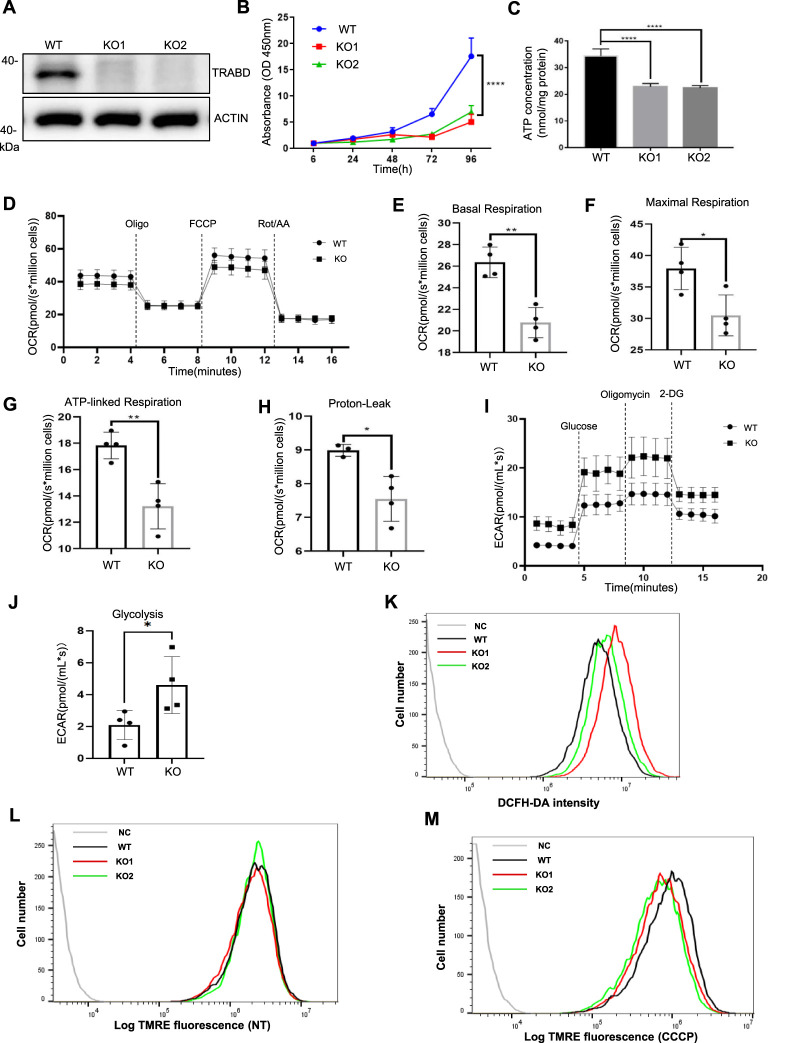
*TRABD* KO perturbs cell growth and mitochondrial function. **(A)** Verification of *TRABD-*KO 293A cells. The whole-cell lysates (WCLs) from WT or *TRABD-*KO cells were analyzed by immunoblotting with anti-TRABD and anti-ACTIN antibodies. **(B)** *TRABD* KO inhibited 293A cell proliferation. The data are presented as the means ± SEM; ****p < 0.0001. **(C)** *TRABD* KO inhibited ATP production in 293A cells. The data are presented as the means ± SEM; ****p < 0.0001. **(D–H)** *TRABD* KO disrupted mitochondrial respiration. Oxygen consumption was recorded at baseline and after the sequential injection of oligomycin, FCCP or antimycin A **(D).** Basal respiration **(E)**, maximal respiration **(F)**, ATP-linked respiration **(G)**, and proton leakage **(H)** were quantified. The data are presented as the means ± SEM; *p < 0.05; **p < 0.01. **(I–J)** *TRABD* KO increased mitochondrial glycolysis. The pH of the culture medium was measured at baseline and after sequential injections of glucose, oligomycin, and 2-deoxy-D-glucose (2-DG) **(I)**. Glycolysis was quantified **(J)**. The data are presented as the means ± SEM; *p < 0.05. **(K)** *TRABD* KO increased intracellular ROS production. Intracellular ROS generation was determined by DCFH-DA in WT and *TRABD-*KO 293A cells. **(L,M)** The mitochondrial membrane potential was determined by TMRE in WT and *TRABD-*KO 293A cells under normal culture **(L)** or CCCP treatment **(M)**. Notably, *TRABD* KO decreased the mitochondrial membrane potential in the presence of CCCP.

To study the function of TRABD in cells, we generated *TRABD* knockout (KO) 293A cells via the CRISPR/Cas9 system. Two knockout 293A single-cell clones were selected and verified by immunoblotting with a homemade anti-TRABD polyclonal antibody (Figure 2A) and genomic sequencing (Supplementary Figure S2A). We observed that *TRABD*-KO 293A cells grew slower than wild-type (WT) 293A cells did, indicating that *TRABD* KO may affect cell proliferation; therefore, we performed a CCK-8 assay to verify and quantify this phenotype. *TRABD* KO significantly inhibited 293A cell proliferation (Figure 2B). We further performed cell cycle analysis via flow cytometry. *TRABD* KO caused significant cell cycle arrest in the G0/G1 and S phases (Supplementary Figure S2B). Mitochondria are the major energy sources in most cells by generating ATP through oxidative phosphorylation. We therefore measured the cellular ATP levels in WT and *TRABD*-KO 293A cells. *TRABD* KO significantly reduced cellular ATP levels (Figure 2C), suggesting that *TRABD* KO reduces mitochondrial oxidative phosphorylation. In 293A *TRABD*-knockdown (KD) cells, we also observed a reduction in ATP levels accompanied by an inhibition of cell proliferation. However, in HeLa cells, *TRABD* KD significantly reduced ATP production but had no effect on cell proliferation (Supplementary Figure S2C–E), suggesting that TRABD primarily regulates ATP production but not cell growth.

We further measured the mitochondrial oxygen consumption rate via Oroboros O2k, a high-resolution respirometer. Compared with WT 293A cells, *TRABD*-KO 293A cells presented decreased respiratory parameters (decreased basal and maximal respiratory capacity) (Figures 2D–F), a trend toward decreased ATP production (Figure 2G), and decreased proton leakage (Figure 2H). The extracellular acidification rate (ECAR) reflects cellular glycolytic activity and complements the oxygen consumption rate (OCR), providing a comprehensive understanding of overall cellular energy metabolism (Schmidt et al., 2021; Yetkin-Arik et al., 2019). To better assess the impact of *TRABD* KO on cellular metabolic function, we measured the ECAR via Oroboros O2k. The results indicated that glycolysis was significantly elevated in *TRABD*-KO cells, as shown in Figures 2I–J. Mitochondria are considered relevant sources of reactive oxygen species (ROS) in living cells, and *TRABD* KO increased the level of cellular ROS, as measured with a DCFH-DA probe (Figure 2K). The mitochondrial membrane potential is an important parameter of mitochondrial function. Although *TRABD* KO had little effect on the mitochondrial membrane potential under normal culture conditions (Figure 2L), *TRABD* KO significantly decreased the mitochondrial membrane potential under carbonyl cyanide m-chlorophenylhydrazone (CCCP; for mitochondrial membrane depolarization) treatment (Figure 2M).

Taken together, these results suggest that TRABD plays essential roles in maintaining normal mitochondrial function.

### 3.3 TRABD is critical for maintaining mitochondrial dynamics

**FIGURE 3:**
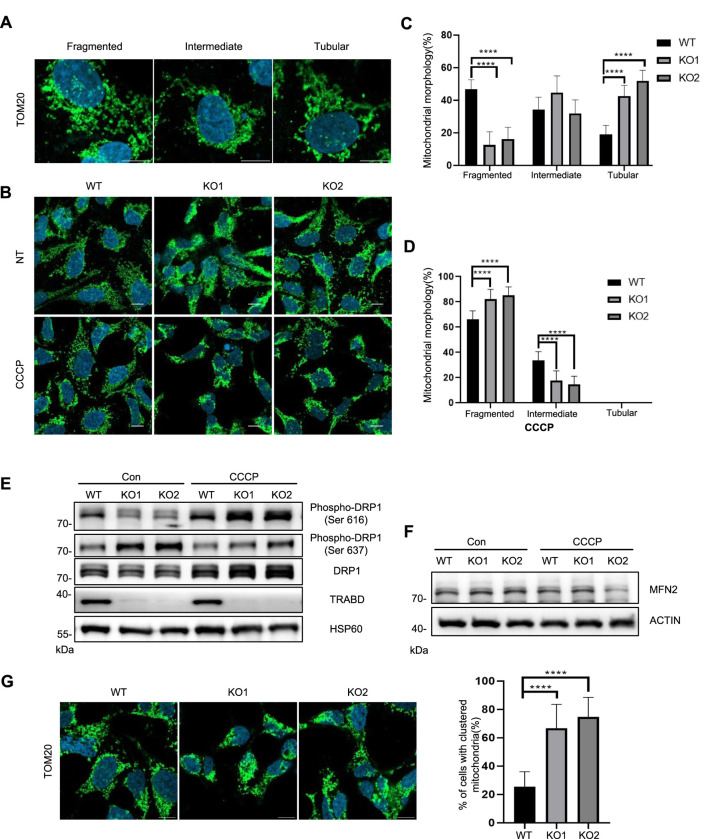
*TRABD* KO affects mitochondrial dynamics. **(A)** Three typical mitochondrial morphologies in WT or *TRABD-*KO 293A cells. The mitochondria were visualized by immunostaining for TOM20. (Scale bar, 10 μm). **(B)** The morphology of mitochondria in WT or *TRABD*-KO 293A cells under normal and CCCP conditions. The mitochondria were visualized by immunostaining for TOM20. (Scale bar, 10 μm). **(C)** Scoring of mitochondrial network morphologies in WT or *TRABD-*KO 293A cells under normal culture conditions. The data are presented as the means ± SEM; ****p < 0.0001. **(D)** Scoring of mitochondrial network morphologies in WT or *TRABD-*KO 293A cells under CCCP treatment. The data are presented as the means ± SEM; ****p < 0.0001. **(E)** Crude mitochondrial fractions from WT or *TRABD-*KO 293A cells cultured with or without CCCP treatment were analyzed by immunoblotting with the indicated antibodies. Notably, *TRABD* KO decreased total DRP1 and pDRP1 (Ser616) but increased pDRP1 (Ser637) under normal culture conditions, whereas *TRABD* KO increased both total DRP1 and pDRP1 (Ser616) under CCCP treatment. **(F)** WT and *TRABD* KO 293A cells were treated with DMSO or CCCP, and whole-cell lysates (WCLs) were analyzed by immunoblotting using the indicated antibodies. **(G)** *TRABD* KO perturbed mitochondrial motility in the presence of CCCP. WT or *TRABD-*KO 293A cells treated with CCCP were fixed and subjected to immunostaining for TOM20, and the cellular distribution of mitochondria was quantified. (Scale bar, 10 μm). The data are presented as the means ± SEM; ****p < 0.0001.

Mitochondrial dynamics, including mitochondrial fusion, fission, and motility, are critical for maintaining mitochondrial homeostasis and enabling cells to adapt to energy demands and other stresses (Wai et al., 2016). We first examined mitochondrial morphology via quantitative imaging analysis, in which mitochondria were classified into three categories according to their fragmentation status: fragmented, intermediate, and tubular (Figure 3A) (Rambold et al., 2011). Under normal culture conditions, *TRABD* KO reduced the number of fragmented mitochondria but increased the number of tubular mitochondria (Figures 3B,C). Interestingly, under CCCP treatment, the numbers of fragmented and intermediate mitochondria in *TRABD*-KO cells increased and decreased, respectively (Figures 3B,D). We conclude that TRABD is important for mitochondrial morphology under basal and CCCP-induced conditions. DRP1 plays critical roles in regulating mitochondrial fission (Wai et al., 2016), and DRP1 activity is controlled by DRP1 phosphorylation. Previous studies have suggested that Ser616 phosphorylation promotes DRP1 activity (Taguchi et al., 2007), whereas Ser637 phosphorylation inhibits DRP1 activity during mitochondrial fission (Chang and Blackstone, 2007). Immunoblotting analysis of crude mitochondrial fractions revealed that *TRABD* KO reduced the levels of both total and Ser616-phosphorylated DRP1 but increased the level of DRP1 phosphorylated at Ser637 in mitochondria under normal culture conditions (Figure 3E). On the other hand, *TRABD* KO increased both total and Ser616-phosphorylated DRP1 in mitochondria under CCCP treatment (Figure 3E). These results are consistent with the mitochondrial fragmentation status in WT and *TRABD*-KO 293A cells under normal culture conditions or after CCCP treatment. Mitofusin 2 (MFN2), a critical regulator of mitochondrial fusion, was examined. The results showed no significant differences in MFN2 levels between WT and KO cells under normal conditions. However, a trend toward reduced was observed MFN2 levels in *TRABD* KO cells following CCCP treatment (Figure 3F). Interestingly, *TRABD* KO also caused perinuclear aggregation of mitochondria in the presence of CCCP (Figure 3G), suggesting that TRABD may also regulate mitochondrial trafficking. Taken together, these results suggest that TRABD plays essential roles in mitochondrial dynamics and motility.

### 3.4 TRABD knockout promotes mitophagy

**FIGURE 4:**
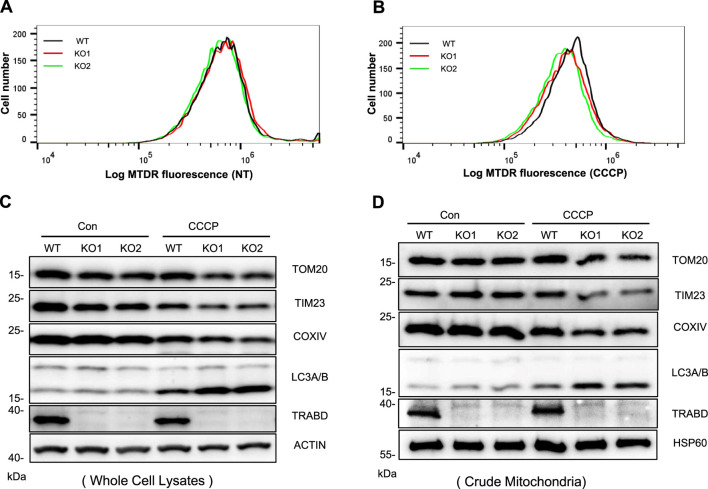
*TRABD* KO promotes mitophagy. **(A)** MTDR was used to determine mitochondrial staining by flow cytometry in WT or *TRABD-*KO 293A cells under normal culture conditions. **(B)** MTDR was used to determine mitochondrial staining by flow cytometry in WT or *TRABD-*KO 293A cells treated with CCCP (30 μM for 6 h). **(C)** WT and *TRABD-*KO 293A cells were treated with DMSO or 30 μM CCCP for 6 h, and whole-cell lysates (WCLs) were analyzed by immunoblotting with the indicated antibodies. **(D)** WT and *TRABD-*KO 293A cells were treated with DMSO or 30 μm CCCP for 6 h, and the crude mitochondrial fractions were analyzed by immunoblotting with the indicated antibodies.

Mitophagy is an important mitochondrial quality control mechanism by which cells clear damaged and aged mitochondria through the autophagy–lysosomal pathway, which is closely related to mitochondrial dynamics and motility (Montava-Garriga and Ganley, 2020). Since TRABD regulates mitochondrial dynamics and motility, we examined the effect of TRABD on mitophagy. We first used MitoTracker Deep Red (MTDR)-based flow cytometry to monitor mitophagy (Mauro-Lizcano et al., 2015). Although *TRABD* KO had little effect on mitophagy under normal culture conditions (Figure 4A), *TRABD* KO augmented CCCP-induced mitophagy (Figure 4B), as the intensity of the MTDR fluorescence signal in *TRABD*-KO cells was lower than that in WT cells, which represents the total number of mitochondria. Furthermore, immunoblotting revealed that *TRABD* KO reduced the levels of the mitochondrial proteins TOM20, TIM23, and COXIV and increased the levels of LC3-II both in whole-cell lysates and in crude mitochondrial fractions under CCCP treatment but not under normal culture conditions (Figures 4C,D). Taken together, these results suggest that *TRABD* KO promotes mitophagy under stress conditions.

### 3.5 TRABD interacts with PGAM5

**FIGURE 5:**
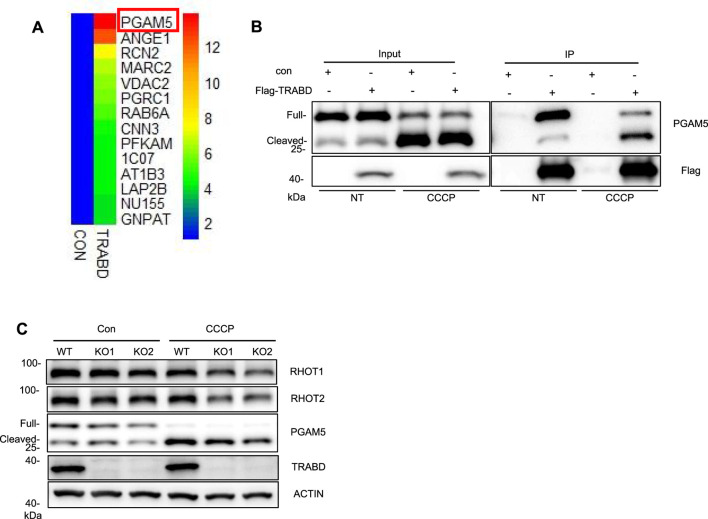
TRABD interacts with PGAM5. **(A)** 293T cells stably expressing HA-EGFP or HA-TRABD were lysed and subjected to immunoprecipitation with an anti-HA antibody, and the precipitates were further analyzed by quantitative mass spectrometry. Among the top fourteen potential TRABD-interacting proteins, PGAM5 had the highest score. **(B)** Verification of the interaction between TRABD and PGAM5. Control 293T or 293T cells expressing FLAG-TRABD were lysed and subjected to immunoprecipitation with an anti-FLAG antibody. The WCLs (input) and precipitates (IP) were analyzed by immunoblotting with anti-PGAM5 or anti-FLAG antibodies. Notably, CCCP treatment caused the cleavage of PGAM5, and TRABD interacted with both full-length and cleaved PGAM5. **(C)** WT and *TRABD-*KO 293A cells were treated with DMSO or 30 μm CCCP for 6 h, and the WCLs were analyzed by immunoblotting with the indicated antibodies.

To screen for proteins that interact with the TRABD, 293T cells stably expressing HA-TRABD or HA-EGFP-MT (a transmembrane domain from a mitochondrial outer membrane protein fused to the carboxyl terminus of EGFP) were subjected to immunoprecipitation experiments with the anti-HA antibody agarose, and the precipitates were analyzed by quantitative mass spectrometry. HA-EGFP-MT was used as a negative control to help identify proteins that specifically interact with TRABD, and PGAM5 was among the top hit proteins (Figure 5A). PGAM5 reportedly localizes to both the inner and outer membranes of mitochondria and is regulated by mitochondrial proteases to generate a cleaved short form of PGAM5 under stress conditions, such as CCCP treatment (Cheng et al., 2021). We verified the interaction between TRABD and PGAM5 under both normal culture conditions and after CCCP treatment via coimmunoprecipitation. The results indicated that TRABD interacts with both the full-length and short forms of PGAM5 under both conditions (Figure 5B). PGAM5 plays critical roles in mitochondrial dynamics and mitophagy. The immunoblotting results revealed that *TRABD* KO reduced the levels of the short form of PGAM5 under CCCP treatment (Figure 5C). Interestingly, the protein levels of Ras homolog family member T1 (RHOT1/Miro1) and Ras homolog family member T2 (RHOT2/Miro2), two mitochondrial Rho GTPases that play critical roles in mitochondrial transport (López‐Doménech et al., 2018), were also reduced by *TRABD* KO under CCCP treatment (Figure 5C). A previous study suggested that PGAM5 regulates mitochondrial transport via RHOT2 (O’Mealey et al., 2017). Taken together, these results suggest that TRABD may regulate mitochondrial dynamics and mitophagy through binding to and regulating PGAM5.

### 3.6 *TRABD* knockout in mice exacerbates ischemia reperfusion-induced renal tubular injury

**FIGURE 6:**
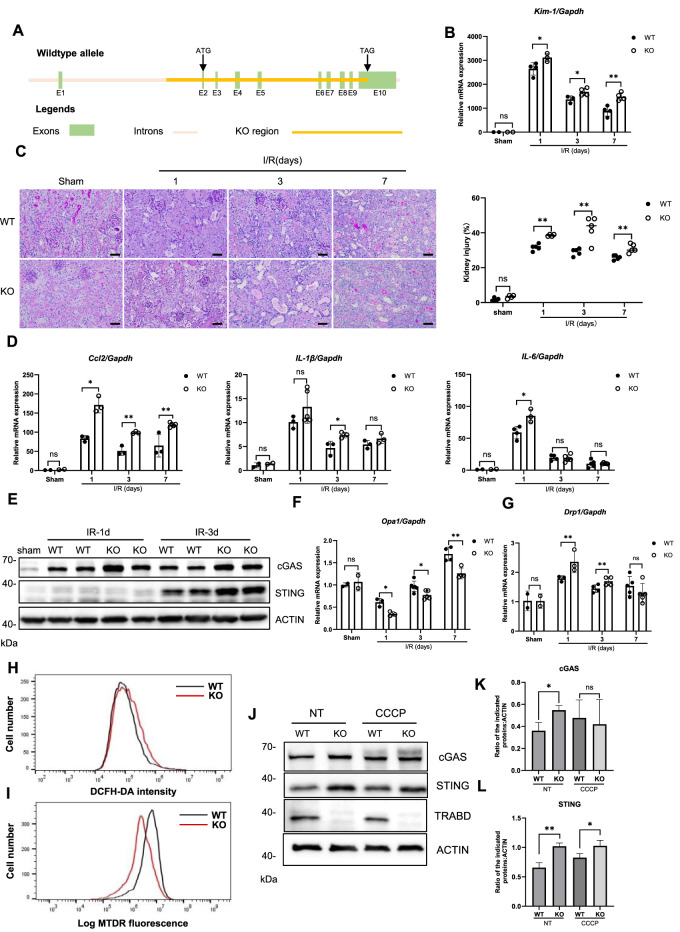
*TRABD* KO exacerbates ischemia reperfusion-induced renal tubular injury. **(A)** Schematic illustration of the strategy used to generate *TRABD-*knockout mice. **(B)** Quantitative RT‒PCR analysis of *Kim-1* mRNA levels in mouse kidneys. **(C)** Structural alterations in tubules after I/R, as illustrated by PAS staining and quantitative analysis (scale bar, 40 μm). **(D)** Quantitative RT‒PCR analysis of *Ccl2, IL-1β, and IL-6* expression in mouse kidneys. **(E)** Immunoblotting analysis of cGAS and STING expression in mouse kidneys. **(F,G)** Quantitative RT‒PCR analysis of *Opa1* **(F)** and *Drp1* **(G)** expression in mouse kidneys. **(H)** *TRABD* KO increased intracellular ROS production. Intracellular ROS generation was determined by DCFH-DA in WT and *TRABD*-KO PTECs. **(I)** The mitochondrial membrane potential was determined by TMRE in WT and *TRABD-*KO PTECs. **(J)** Immunoblotting analysis of cGAS and STING expression in PTECs under normal conditions or treated with CCCP. **(K,L)** Quantification of the relative expression levels of the indicated proteins. All data are presented as the means ± SEM; ns, not significant. *P < 0.05, **P < 0.01.

TRABD is ubiquitously expressed in humans and mice according to the gene expression database. To explore the function of TRABD in mammals *in vivo*, we generated *TRABD* knockout mice via the CRISPR/Cas9-based technique, in which the entire coding region of TRABD in the mouse genome was deleted (Figure 6A). The successful knockout of *TRABD* in mice was verified by genotyping PCR and sequencing (Supplementary Figure S3A,B). *TRABD*-KO mice are viable and fertile without overt defects. The immunoblotting results verified that *TRABD* KO efficiently abolished TRABD protein expression in mouse kidneys. As *TRABD* KO results in pronounced dysregulation of mitochondrial dynamics and mitophagy, mainly under CCCP treatment, we hypothesized that TRABD may play critical roles in stress conditions, such as renal tubular injury, in mice. To test this hypothesis, we used an ischemia reperfusion-induced renal tubular injury mouse model. Littermates WT and *TRABD*-KO mice were subjected to 30 min of unilateral renal ischemia or sham surgery, and renal tubular injury and related gene expression were measured after 1, 3, and 7 days of reperfusion. *TRABD* KO resulted in increased *Kim-1* and *Ngal* gene expression in injured kidneys at all three time points post-I/R (Figure 6B; Supplementary Figure S3C). Histological analysis of renal cortical tissues revealed that *TRABD* KO significantly increased the severity of renal tubular injury at all three time points post-I/R, as demonstrated by the quantification of the detachment and death of tubular cells and the loss of brush borders (Figure 6C). Moreover, TUNEL staining showed a significantly higher number of apoptotic cells in the kidney tissue of the *TRABD* KO compared to the WT (Supplementary Figure S3D–E), confirming that TRABD deficiency exacerbates I/R-induced renal cell apoptosis. Furthermore, *TRABD* KO increased the expression of the *Ccl2, IL-1β*, and *IL-6* genes in injured renal tissues, suggesting more severe inflammation in the injured kidneys of *TRABD*-KO mice than in those of WT mice post I/R (Figure 6D). Moreover, the evaluation of renal function showed that *TRABD* KO mice had significantly higher levels of blood urea nitrogen (BUN) and serum creatinine (Scr) compared to WT mice (Supplementary Figure S3F–G). However, the unilateral renal ischemia and reperfusion model resulted in a less pronounced increase in these parameters compared to the bilateral ischemia and reperfusion model, likely due to the compensatory function of the contralateral kidney.

Mitochondrial dysfunction and subsequent activation of the mtDNA-cGAS-STING pathway are among the key features of ischemia reperfusion kidney injury (Hopfner and Hornung, 2020; Maekawa et al., 2019). Immunoblotting analysis of mouse renal tissues revealed that *TRABD* KO increased the protein levels of both cGAS and STING at 1 and 3 days post-I/R (Figure 6E), suggesting that *TRABD* KO increased the severity of mitochondrial damage and the release of mtDNA. Furthermore, *TRABD* KO decreased *Opa1* expression and increased *Drp1* gene expression in injured renal tissues, suggesting the presence of more fragmented mitochondria in the renal tissues of *TRABD*-KO mice after I/R (Figure 6F‒G). We also isolated and cultured PTECs from both WT and *TRABD*-KO mouse kidney tissues. Flow cytometry analysis revealed that cellular ROS levels were increased, and that the mitochondrial membrane potential was decreased in *TRABD*-KO PTECs (Figures 6H,I). Immunoblotting revealed that the protein levels of both cGAS and STING were increased in *TRABD*-KO PTECs under both normal conditions and CCCP treatment (Figures 6J–L). These results suggest that *TRABD* KO disrupts mitochondrial homeostasis and function in PTECs.

Taken together, these results suggest that *TRABD* KO exacerbates kidney injury possibly through promoting mitochondrial fragmentation and damage after I/R.

## 4 Discussion

In this study, we characterized TRABD as a novel outer mitochondrial membrane protein that is essential for maintaining mitochondrial homeostasis and normal functions, possibly through binding to PGAM5. Depletion of *TRABD* in HEK293A cells severely impairs mitochondrial function and causes reductions in mitochondrial respiration and ATP production and an increase in ROS production. Under CCCP treatment, the depletion of *TRABD* promoted mitochondrial fission and mitophagy but inhibited the transport of mitochondria. We further revealed a protective role of TRABD in I/R-induced renal tubular injury.

Mitochondrial dynamics play critical roles in maintaining mitochondrial homeostasis and function and can be regulated by outer mitochondrial membrane proteins. Our results suggest that TRABD may also regulate mitochondrial dynamics. Interestingly, depletion of TRABD had opposite effects on mitochondrial fragmentation under normal culture conditions and under CCCP treatment. Under normal culture conditions, *TRABD*-KO cells presented more tubular mitochondria and increased DRP1 phosphorylation at Ser637. However, in the presence of CCCP, the depletion of *TRABD* may promote mitochondrial fission and cause more fragmentation of mitochondria in cells, and DRP1 phosphorylation at Ser616 is elevated in *TRABD*-KO cells. In addition, MFN2 exhibits a decreasing trend in TRABD KO cells following CCCP treatment. The above findings suggest that the effect of TRABD on mitochondrial morphology is associated with changes in DRP1 and MFN2. We speculate that TRABD may regulate mitochondrial dynamics through multiple mechanisms dependent on different stress conditions. PGAM5 is a multifunctional protein that regulates mitochondrial dynamics and mitophagy (Nag et al., 2023; Sugo et al., 2018; Yu et al., 2020). TRABD interacts with PGAM5 and may regulate its function in mitochondria. PGAM5 may also regulate TRABD as a protein phosphatase. RHOT1/2 regulates mitochondrial transport in different cell types (López‐Doménech et al., 2018), which is downregulated by *TRABD* KO under CCCP treatment. Furthermore, a correlation between PGAM5 and RHOT2 was revealed in a recent study (Cheng et al., 2021). PGAM5 plays a pivotal role in various cellular processes, including mitophagy, apoptosis, and mitochondrial dynamics (Cheng et al., 2021). In this study, we observed that TRABD consistently interacts with PGAM5 under both normal and CCCP-treated conditions. We hypothesize that TRABD may bind to and stabilize PGAM5, and its deficiency could disrupt the composition of the PGAM5 complex or alter its subcellular localization. These changes could potentially impair PGAM5’s activity or affect its specificity for certain targets. Although we observed a reduction in both PGAM5 and RHOT1/RHOT2 levels in TRABD-deficient 293A cells treated with CCCP, the complex function of PGAM5 makes it difficult to conclusively establish a direct causal relationship between this reduction and mitochondrial autophagy or dynamics. Furthermore, under CCCP treatment conditions, TRABD deficiency promotes DRP1-S616 phosphorylation and an increase in total DRP1 levels, thereby enhancing mitochondrial fission and fragmentation, processes typically associated with elevated mitophagy. Under stress conditions, PGAM5’s role may be more complex. Thus,the detailed regulatory mechanisms require further in-depth investigation.

Global knockout of TRABD in mice does not cause obvious phenotypes, suggesting that TRABD is not essential in mice under normal conditions or that its function can be compensated for by other proteins in mice. However, this is not surprising among mitochondrial proteins. For example, mice lacking PGAM5 or FUNDC1 are also viable and fertile under normal conditions but exhibit severe phenotypes under stress conditions such as I/R-induced tissue injuries (Zhang et al., 2017; Zhu et al., 2021). We adopted an I/R-induced renal tubular injury mouse model and reported that depleting TRABD significantly exacerbates renal tubular injury by promoting mitochondrial fragmentation and damage. These results suggest that TRABD plays a protective role against I/R-induced renal tubular injury.

The TRABD belongs to the Tiki/TraB family of potential metalloproteases that are conserved among the animal kingdom (Bazan et al., 2013). Interestingly, the plant homologs of Tiki/TraB proteins were recently identified as TRB1/2. TRB1/2 are localized in ER‒mitochondrion contact sites and promote mitophagy by binding to ATG8 via their ATG8-binding motif (AIM) (Li et al., 2022). The AIMs in TRB1/2 are not conserved in TRABD. Furthermore, our study suggested that TRABD may negatively regulate mitophagy, as depletion of TRABD promoted mitophagy in response to CCCP treatment. These results suggest that TRABD and TRB1/2 may have distinct functions in mitochondria despite both being localized on the outer mitochondrial membrane. TIKI proteins are putative metalloproteases located on the plasma membrane via GPI anchors. Whether TRABD and TRB1/2 function as proteases on the outer mitochondrial membrane remains to be investigated.

Our study not only confirms the previously reported mitochondrial localization of TRABD but also offers new insights into its regulation, underlying mechanisms, and physiological roles. We demonstrate that TRABD impacts mitochondrial morphology, membrane potential, respiration, glycolysis, and mitophagy, with significant variation across different cell types. Moreover, we reveal that TRABD’s precise localization relies on its C-terminus, with its topology showing the N-terminus facing the cytoplasm and the C-terminus positioned in the matrix. We also identify a novel interaction partner, PGAM5, distinct from the MFN2/MIGA2/PLD6 complex. Notably, our research highlights TRABD’s critical protective role in renal tubular epithelial cells during ischemia reperfusion, where it preserves mitochondrial homeostasis and prevents the activation of the cGAS-STING immune pathway. In summary, our findings deepen the understanding of TRABD’s cell-type-specific functions, uncover a new molecular mechanism involving PGAM5, and establish its essential role in mitochondrial quality control and kidney protection.

## Data Availability

The datasets presented in this study can be found in online repositories. The names of the repository/repositories and accession number(s) can be found in the article/Supplementary Material.
