# Supplementary figures and images for: TRABD maintains mitochondrial homeostasis and protects against ischemia reperfusion-induced renal tubular injury
Source: Front Cell Dev Biol. 2025 Jul 24;13:1619339. doi: 10.3389/fcell.2025.1619339 (PMC12329661; doi:10.3389/fcell.2025.1619339)

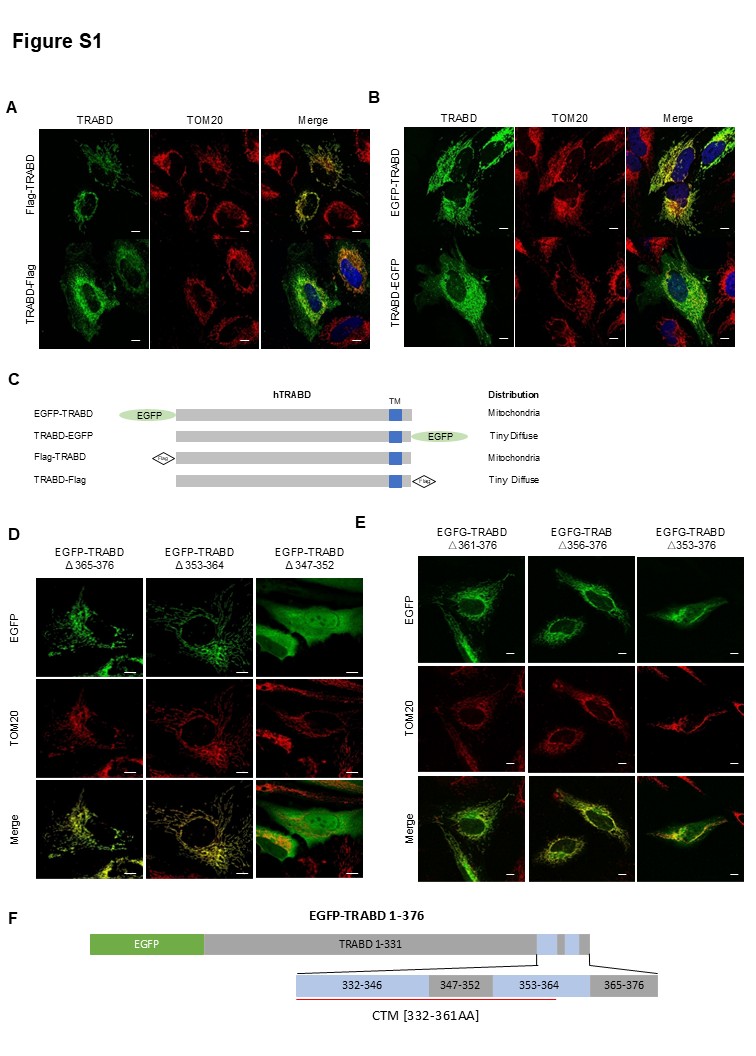

Supplement: Supplementary file 1 [file Image1.jpg]
